# Calciphylaxis epidemiology, risk factors, treatment and survival among French chronic kidney disease patients: a case-control study

**DOI:** 10.1186/s12882-020-01722-y

**Published:** 2020-02-26

**Authors:** Raphaël Gaisne, Morgane Péré, Victorio Menoyo, Maryvonne Hourmant, David Larmet-Burgeot

**Affiliations:** 1 Department of Nephrology and Immunology, Institute of Transplantation Urology and Nephrology, Centre Hospitalier Universitaire de Nantes, Nantes, France; 2 Service de Néphrologie et Immunologie Clinique, Centre Hospitalier Universitaire de Nantes, 30, bd Jean Monnet 44093, Nantes, Cedex 01 France; 3 Biostatistician, Direction de la Recherche, Plateforme de Méthodologie et Biostatistiques, Centre Hospitalier Universitaire de Nantes, Nantes, France; 4 ECHO Department of Medical Information, Nantes, France; 5 Department of Nephrology, Centre Hospitalier de Saint Nazaire, St Nazaire, France

**Keywords:** Calcific uremic arteriolopathy, Calciphylaxis, Case-control study, ESRD, Vitamin K antagonist

## Abstract

**Background:**

Calcific Uremic Arteriolopathy (CUA) is a rare disease, causing painful skin ulcers in patients with end stage renal disease. Recommendations for CUA management and treatment are lacking.

**Methods:**

We conducted a retrospective cohort study on CUA cases identified in western France, in order to describe its management and outcome in average clinical practices. Selection was based on the Hayashi diagnosis criteria (2013) extended to patients with eGFR < 30 mL/min/1.73m^2^. Dialyzed CUA cases were compared with 2 controls, matched for age, gender, region of treatment and time period.

**Results:**

Eighty-nine CUA cases were identified between 2006 and 2016, including 19 non dialyzed and 70 dialyzed patients. Females with obesity (55.1%) were predominant. Bone mineral disease abnormalities, inflammation and malnutrition (weight loss, serum albumin decrease) preceded CUA onset for 6 months. The multimodal treatment strategy included wound care (98.9%), antibiotherapy (77.5%), discontinuation of Vitamin K antagonists (VKA) (70.8%) and intravenous sodium thiosulfate (65.2%). 40.4% of the patients died within the year after lesion onset, mainly under palliative care. Surgical debridement, distal CUA, localization to the lower limbs and non calcium-based phosphate binders were associated with better survival. Risks factors of developing CUA among dialysis patients were obesity, VKA, weight loss, serum albumin decrease or high serum phosphate in the 6 months before lesion onset.

**Conclusion:**

CUA involved mainly obese patients under VKA. Malnutrition and inflammation preceded the onset of skin lesions and could be warning signs among dialysis patients at risk.

**Trial registration:**

ClinicalTrials.gov identifier NCT02854046, registered August 3, 2016.

## Background

Calcific uremic arteriolopathy (CUA), also called calciphylaxis, is a rare but devastating disease involving patients with end stage renal disease (ESRD). CUA causes painful skin lesions that evolve to ulcerative lesions at risk of superinfection and sepsis [1], with a poor prognosis. One year survival rates vary between 45 to 55% [2–4]. CUA management lacks strong recommendations [5] and therefore is heterogeneous. Reported risk factors of CUA are female sex, obesity, diabetes mellitus, vitamin K antagonists (VKA) and ESRD [6]. Dysregulation of calcium-phosphate metabolism also participates to its development. Histopathological findings of skin lesions mostly associate thromboses and vessel calcifications [7]. We decided to conduct the first study on CUA in the French population. Our main objective was to describe diagnosis management, treatment and outcome of CUA in ESRD and stage 4–5 CKD patients. Secondary objectives were to analyze risk factors of developing calciphylaxis and influencing patient survival in the dialyzed cohort.

## Materials and methods

### Study patients

We first conducted a retrospective cohort study and secondly a case control analysis. The nephrologists from Western France were asked to report their patients diagnosed for CUA. Patients were also identified by searching the MEDIAL dialysis regional data base. Inclusion and non-inclusion criteria checking and collection of the data in the medical records were performed by the investigators in the center of care of each case.

Hayashi [8] criteria were used for diagnosis: chronic hemodialysis or estimated Glomerular Filtration Rate (eGFR) below 15 mL/min/1.73m^2^, more than two painful non-treatable skin ulcers with concomitant painful purpura and localization of skin ulcers on the trunk, extremities or penis with concomitant painful purpura. Typical histopathological findings (necrosis and ulceration of the skin with calcification of the tunica media and internal elastic membrane of small to medium-sized arterioles of dermis and subcutaneous fat) can replace a clinical feature.

Inclusion criteria were: CUA according to Hayashi criteria, onset of cutaneous lesions between 1st January 2006 and 31th December 2016, patients > 18 yo. Patients with eGFR between 15 and 30 mL/min/1.73m^2^ (CKD EPI formula) (the serum creatinine at onset of CUA was considered) were also included if all other inclusion criteria were met. CUA was eliminated if a differential diagnosis seemed more likely or was confirmed by skin biopsy, or in case of severe atherosclerotic vascular disease in the wound area. CUA patients under hemodialysis or peritoneal dialysis at onset of CUA lesions were assigned to the dialysis group, while the others to the non dialysis group.

### Controls selection

In order to explore risk factors of CUA among dialyzed patients, each CUA dialysis patient was matched with two controls identified in the REIN registry of treated ESRD in France. Matching criteria were: gender, age (± 2 years), treatment by hemodialysis in the same geographical area and at the same time of CUA diagnosis in the case. Among the potential controls for each case, two were randomly and anonymously selected. After checking the absence of diagnosis of CUA, collection of the data in the medical records of the control patients was performed by the investigator directly in the center of care of the patient.

### Study data

The analyzed data were demographic, history of kidney disease, cardiovascular comorbidities and other previously reported risk factors of CUA. Thrombophilia was defined as acquired or congenital antithrombin/protein C/protein S deficiency, antiphospholipid syndrome, activated protein C resistance, prothrombin mutation. “Onset date” of CUA was the date where typical skin lesions were mentioned in medical records. Laboratory data at onset were recorded as well as the worst values within the 6 months before diagnosis. As intact Parathyroid hormone (iPTH) measurement kits were different between laboratories, we normalized iPTH with the upper limit of the normal range for each laboratory. Medications and dialysis parameters were recorded. For each CUA case, clinical presentation with lesion distribution defined as proximal (extremities proximal to knees and elbows, trunk, breast and penis) and/or distal (extremities distal to knees and elbows), evolution, diagnosis and treatment methods and outcomes were collected.

A written consent form was given to each patient, except for deceased patients and loss of follow-up patients. The study was approved by the ethics committee of the Nantes University Hospital. All data collected were de-identified.

### Statistical analyses

Frequency of categorical variables, median and interquartile range (IQR) for non-normally distributed variables were reported. Survival curves were determined using the Kaplan-Meier method. Survival analysis using Cox models were used to determine survival predictors. In the group of CUA patients treated by dialysis, univariate conditional logistic regression analysis on matched case-controls was performed. Variables with *p* < 0.20 were included in the multivariate logistic regression analysis. Non dialyzed CUA patients were not included in the risk factor analysis because of the lack of controls for those patients.

All analyses were performed using the SAS program (version 9.4) (SAS Institute Inc., Cary, NC). Statistical significance was set as *p* < 0.05. The study protocol was pre-registered on clinicaltrials.gov under the number NCT02854046.

## Results

**Fig. 1:**
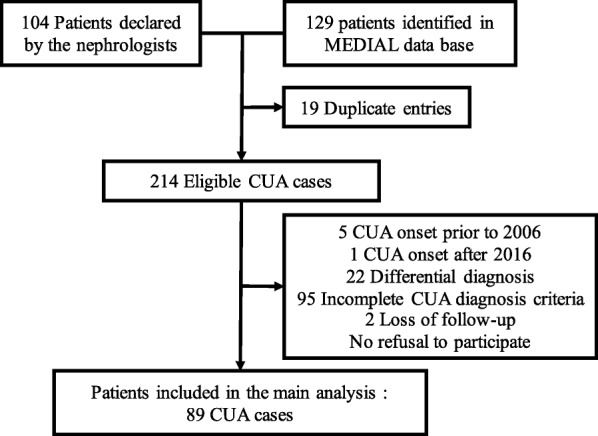
Flow chart of selection of Calcific Uremic Arteriolopathy cases

Two hundred fourteen eligible cases were identified (Fig. 1). Ninety-five patients with incomplete diagnosis criteria and 22 with a differential diagnosis were excluded (Table S1). Eighty-nine cases of CUA were finally included, 74% diagnosed between 2013 and 2016.

### Baseline characteristics (Table 1)

75.3% of cases were hemodialyzed. Among the 19 patients with stage 4–5 chronic kidney disease (CKD), median eGFR was 10.3 ml/min/1.73m^2^ (IQR 8.4–13.0) and median blood urea nitrogen was 31 mmol/L (IQR 20–47). CUA patients were obese (median Body Mass Index (BMI) 31 kg/m^2^) and had a recent median weight loss of 3.5 kg. In dialyzed and non dialyzed CUA cases, the main causes of CKD were respectively diabetes-associated nephropathy (25.7 and 26.3%), hypertension-associated nephropathy (22.9 and 0%), hypertension and diabetes-associated nephropathy (15.7 and 21.1%) and glomerular nephropathy (10.0 and 26.3%). 5 CUA patients only had proven thrombophilia.

**Table 1:** Demographic data of CUA cases and the matched dialysis controls

Parameter	N	Total CUA cases	N	Non dialysis cases	N	Dialysis cases	N	Dialysis controls	*P* Value
Age (years)	89	70 (63–78)	19	71 (58–79)	70	70 (64–78)	140	69 (63–77)	
Females	89	57 (64.0%)	19	8 (42.1%)	70	49 (70.0%)	140	98 (70.0%)	
BMI (kg/m^2^)	89	31.0 (25.3–37.3)	19	34.6 (28.6–39.2)	70	30.7 (24.5–37.1)	137	25.1 (21.6–28.6)	< 0.001
Overweight (BMI 25–30 kg/m2)		20 (22.5%)		6 (31.6%)		14 (20.0%)		40 (29.2%)	
Obesity (BMI 30–40 kg/m2)		33 (37.1%)		10 (52.6%)		23 (32.9%)		26 (19.0%)	
Severe obesity (BMI > 40 kg/m2)		16 (18.0%)		3 (15.8%)		13 (18.6%)		3 (2.2%)	
Loss of weight within 6 months before diagnosis (kg)	82	3.5 (0.5–7.0)	16	7.0 (3.0–15.9)	66	3.0 (0.0–6.0)	134	0.0 (−1.1–1.5)	< 0.001
CKD stage (eGFR)	89		19		70		140		
CKD Stage 4 (15–30 mL/min/1.73m^2^)		4 (4.5%)		4 (21.0%)					
CKD Stage 5 (< 15 mL/min/1.73m^2^)		15 (16.8%)		15 (70.0%)					
CKD stage 5 under Hemodialysis		67 (75.3%)				67 (95.7%)		140 (100%)	
CKD stage 5 under PD		3 (3.4%)				3 (4.2%)			
CAD	89	41 (46.1%)	19	6 (31.6%)	70	35 (50.0%)	140	50 (35.7%)	0.047
Heart failure	89	51 (57.3%)	19	11 (57.9%)	70	40 (57.1%)	140	34 (24.3%)	< 0.001
Stroke	89	14 (15.7%)	19	3 (15.8%)	70	11 (15.7%)	140	25 (17.9%)	0.70
PAD with symptoms	89	35 (39.3%)	19	4 (21.1%)	70	31 (44.3%)	140	40 (28.6%)	0.02
Diabetes mellitus	89	60 (67.4%)	19	17 (89.5%)	70	43 (61.4%)	140	56 (40.0%)	0.003
Arterial Hypertension	89	85 (95.5%)	19	18 (94.7%)	70	67 (95.7%)	140	121 (86.4%)	0.04
Hypercholesterolemia	89	52 (58.4%)	19	11 (57.9%)	70	41 (58.6%)	140	87 (62.1%)	0.61
History of smoking	89	20 (22.5%)	19	6 (31.6%)	70	14 (20.0%)	137	23 (16.8%)	0.57
Parathyroidectomy	89	4 (4.5%)	19	0 (0.0%)	70	4 (5.7%)	140	8 (5.71%)	1
History of pathologic fracture	89	19 (21.3%)	19	2 (10.5%)	70	17 (24.3%)	140	17 (12.1%)	0.02
Progressive cancer	89	9 (10.1%)	19	2 (10.5%)	70	7 (10.0%)	140	14 (10.0%)	1
Hepatobiliary disease	89	15 (16.9%)	19	2 (10.5%)	70	13 (18.6%)	140	16 (11.4%)	0.16
Chronic alcoholism	89	7 (7.9%)	19	2 (10.5%)	70	5 (7.1%)	140	6 (4.3%)	0.51
Connective tissue disease	89	7 (7.9%)	19	2 (10.5%)	70	5 (7.1%)	140	5 (3.6%)	0.31
Thrombophilia	89	5 (5.6%)	19	0 (0.0%)	70	5 (7.1%)	140	8 (5.7%)	0.76

Median (IQR) or N (%). *P*-Value of comparison of Dialysis Cases with Dialysis controls. *ADPKD* autosomal dominant polycystic kidney disease, *BMI* body mass index, *CAD* coronary artery disease, *CKD* chronic kidney disease, *CUA* calcific uremic arteriolopathy, *PAD* peripheral artery disease, *PD* peritoneal dialysis

### Laboratory findings

Adjusted serum calcium, serum phosphate and normalized iPTH were significantly higher in dialyzed CUA patients than in matched dialyzed controls at lesion onset and in the six preceding months (Table 2). Malnutrition preceded CUA onset, with a median albumin decrease of 2.7 g/L within the 6 months before onset and C-reactive protein (CRP) was high at both times.

**Table 2:** Laboratory parameters measured at onset of CUA and within 6 months before diagnosis (most pejorative value) in CUA and paired dialysis controls

Biological parameter	Recommended range^a^	N	Total CUA cases	N	Non dialysis cases	N	Dialysis cases	N	Dialysis controls	*P* Value
At onset of lesions										
Total serum calcium (mmol/L)	2.10–2.60	89	2.25 (2.12–2.35)	19	2.25 (2.20–2.34)	70	2.23 (2.11–2.38)	138	2.18 (2.08–2.30)	0.04
Adjusted serum calcium (mmol/L)	2.10–2.60	88	2.50 (2.33–2.60)	18	2.51 (2.43–2.68)	70	2.48 (2.31–2.58)	136	2.26 (2.16–2.40)	< 0.001
Serum phosphate (mmol/L)	0.8–1.5	89	1.89 (1.50–2.34)	19	1.98 (1.64–2.60)	70	1.87 (1.46–2.29)	138	1.42 (1.13–1.87)	< 0.001
Calcium phosphate product (mmol^2^/L^2^)		88	4.35 (3.29–5.23)	18	4.59 (3.70–5.74)	70	4.21 (3.19–5.22)	138	3.18 (2.50–4.01)	< 0.001
iPTH (pg/mL)	150–600	87	260 (114–605)	17	115 (83–488)	70	336 (141–605)	136	272 (157–466)	0.20
Normalized iPTH (N)	2–9	87	5.3 (2.1–11.1)	17	2.2 (1.6–10.6)	70	5.3 (2.5–11.1)	136	4.4 (2.4–8.4)	0.04
iPTH outside of target range between 2 and 9 fold normal range		87	51 (58.6%)	17	14 (73.7%)	70	37 (52.9%)	136	58 (42.6%)	0.16
25-Hydroxyvitamin D (ng/mL)	> 30	68	28.5 (17.9–40.0)	13	24.0 (10.0–34.0)	55	29.9 (18.0–40.0)	101	34.1 (22.0–47.6)	0.18
Serum Albumin (g/L)	35–45	88	30.9 (28.0–34.0)	18	30.4 (23.9–34.3)	70	31.5 (28.0–34.0)	136	37.0 (33.3–39.2)	< 0.01
Serum Albumin variation between diagnosis and 6 months before (g/L)		80	−2.7 (−5.0; 1.6)	12	−3.8 (−8.5; −0.5)	68	−2.7 (−5.0; 2.0)	134	1.3 (0.0; 4.0)	< 0.001
CRP (mg/L)	< 5	87	29.0 (8.0–72.0)	17	34.0 (17.5–72.0)	70	23.5 (6.6–64.0)	135	4.2 (1.0–13.8)	< 0.001
Hemoglobin (g/dL)	10–11.5	89	10.4 (9.7–11.6)	19	10.1 (9.7–11.7)	70	10.6 (9.6–11.5)	136	11.2 (10.1–12.0)	0.007
Worst value within 6 months before onset of CUA										
Total serum calcium (mmol/L)	2.10–2.60	86	2.34 (2.17–2.47)	17	2.33 (2.29–2.38)	69	2.35 (2.17–2.48)	137	2.28 (2.20–2.37)	0.30
Adjusted serum calcium (mmol/L)	2.10–2.60	81	2.54 (2.37–2.68)	13	2.54 (2.41–2.71)	68	2.54 (2.30–2.67)	135	2.43 (2.31–2.52)	0.05
Serum phosphorus (mmol/L)	0.8–1.5	86	2.16 (1.75–2.57)	17	2.00 (1.55–2.11)	69	2.25 (1.87–2.70)	137	1.72 (1.44–2.15)	< 0.001
Calcium phosphate product (mmol^2^/L^2^)		86	4.81 (4.24–6.02)	17	4.30 (3.57–4.82)	69	5.20 (4.44–6.50)	137	3.94 (3.26–4.88)	< 0.001
iPTH (pg/mL)	150–600	75	355 (148–710)	10	331 (210–580)	65	435 (148–710)	128	342 (173–526)	0.10
Normalized iPTH (N)	2–9	75	7.3 (3.3–12.2)	10	6.5 (4.5–12.1)	65	7.3 (3.3–12.2)	128	5.3 (2.8–8.5)	0.02
iPTH outside of target value between 2 and 9 fold normal range		75	56 (62.9%)	10	15 (79.0%)	65	41 (63.1%)	128	59 (46.1%)	0.01
25-Hydroxyvitamin D (ng/mL)	> 30	59	29.0 (15.0–42.8)	9	13.0 (9.0–25.0)	50	30.0 (18.0–43.0)	101	36.1 (20.4–48.0)	0.32
Serum Albumin (g/L)	35–45	81	33.0 (29.0–37.0)	13	32.9 (25.0–37.5)	68	33.5 (29.0–37.0)	135	34.0 (32.0–38.0)	0.049
CRP (mg/L)	< 5	80	39.5 (14.0–79.6)	13	32.0 (5.9–56.0)	67	46.0 (14.1–79.9)	133	13.4 (4.5–40.2)	0.004

Median (IQR) or N (%). *P*-Value of comparison between dialysis cases and dialysis controls. *CRP*, C-reactive protein; *iPTH*, intact parathyroid hormone ^a^According to KDIGO clinical practice guideline for the diagnosis, evaluation, prevention, and treatment of chronic kidney disease-mineral and bone disorder (CKD-MBD) Kidney Int Suppl 2009; 113: S1–S130. Adjusted serum calcium level was calculated using the following formula: [serum calcium (mmol/L) + 0,025 (40-Albumin)]

### Medications at CUA lesion onset

Active and native vitamin D were not significantly more prescribed in CUA patients, nor statin and cinacalcet (Table 3). Calcium-based phosphate binders were more frequently administered in CUA patients; 71.9% were under VKA. The median time between VKA introduction and onset of CUA was 3.2 years (IQR 1.8–6.6) and was shorter for dialyzed vs non dialyzed patients (2.6 years (IQR 1.3–5.7) vs 6.5 years (IQR 4.6–8.6)). The median time between dialysis initiation and CUA was 1.4 years (IQR 0.3–3.7). Median dialysis vintage among the control patients, estimated between the beginning of dialysis and the time of onset of CUA in the matched case, was 2.2 years (IQR 0.7–5.1). Median dialysis dose (eKt/V) was significantly lower for CUA cases compared to controls.

**Table 3:** Medications at time of diagnosis of CUA in cases and matched dialysis controls

Treatment	N	Total CUA cases	N	Non dialysis cases	N	Dialysis cases	N	Dialysis controls	*P* Value
25-hydroxyvitamin D	89	53 (59.6%)	19	11 (57.9%)	70	42 (60.0%)	137	89 (65.0%)	0.48
Active vitamin D	89	19 (21.4%)	19	3 (15.8%)	70	16 (22.9%)	137	31 (22.6%)	0.97
Calcium-based phosphate binders	89	47 (52.8%)	19	6 (31.6%)	70	41 (58.6%)	137	60 (43.8%)	0.04
Non calcium-based phosphate binders	89	47 (52.8%)	19	4 (21.1%)	70	43 (61.4%)	137	76 (55.6%)	0.41
Sevelamer	89	37 (41.6%)	19	4 (21.1%)	70	33 (47.1%)	137	58 (41.4%)	0.43
Lanthanum carbonate	89	12 (13.5%)	19	1 (5.3%)	70	11 (15.7%)	137	19 (13.6%)	0.68
Cinacalcet	89	17 (19.1%)	19	1 (5.3%)	70	16 (22.9%)	136	26 (19.1%)	0.53
Betablocker	89	54 (60.7%)	19	15 (79.0%)	70	39 (55.7%)	137	65 (47.5%)	0.26
Insulin therapy	89	35 (39.3%)	19	8 (42.1%)	70	27 (38.6%)	138	34 (24.6%)	0.04
Vitamin K Antagonist	89	64 (71.9%)	19	11 (57.9%)	70	53 (75.7%)	138	37 (26.8%)	< 0.001
Fluindione	64	44 (68.8%)	11	6 (54.6%)	53	38 (71.7%)	37	18 (48.7%)	
Warfarin	64	17 (26.6%)	11	3 (27.7%)	53	14 (26.4%)	37	16 (43.2%)	
Corticosteroids	89	10 (11.2%)	19	2 (10.5%)	70	8 (11.4%)	138	16 (11.6%)	0.97
Statin	89	46 (51.7%)	19	9 (47.4%)	70	37 (52.7%)	138	71 (51.5%)	0.85
ESA	89	72 (80.9%)	19	9 (47.4%)	70	63 (90.0%)	137	106 (77.4%)	0.03
Iron therapy	89	63 (70.8%)	19	8 (42.1%)	70	55 (78.6%)	137	102 (74.4%)	0.51
ACEi/ARB	89	32 (36.0%)	19	8 (42.1%)	70	24 (34.3%)	138	43 (31.2%)	0.65
Hemodialysis parameters									
HD					67	43 (64.2%)	138	91 (65.9%)	
HDF					67	47 (34.1%)	138	24 (35.8%)	
eKt/V					62	1.40 (1.06–1.67)	131	1.53 (1.33–1.82)	< 0.001
Citrate					67	10 (14.3%)	138	20 (14.3%)	1.00

Median (IQR) or N (%). *P*-Value of comparison of Dialysis Cases with Dialysis controls. *ACEi/ARB* angiotensin converting enzyme inhibitor/angiotensin receptor blocker; *ESA* erythropoiesis-stimulating agent, *HD* hemodialysis, *HDF* hemodiafiltration

### Clinical presentation

Fifty-nine CUA cases (66.2%) had a triggering event within the 3 months before onset. Twenty-eight cases (31.5%) had a local trauma, including physical trauma (21%), subcutaneous injection of heparin (25%) or insulin (43%) or both (11%). Thirty-five cases (39.3%) had a hypovolemia episode, including sepsis (29%), general anesthesia (11%), severe intradialytic hypotension (11%), acute heart failure (11%), severe nephrotic syndrome (9%), hemorrhage (5.7%) and multifactorial causes (23%). The same proportion of triggering event was found in dialyzed cases than in non-dialyzed cases (local trauma 30% vs 36.8%, episode of hypovolemia 38.6% vs 42.1% respectively).

Thirty-six patients (40.5%) suffered from a proximal-type CUA, while 26 (29.2%) had a distal-type, and 27 (30.3%) both proximal and distal. Lower limbs were involved in most of the patients (86.5%), especially under the knees (34.8%), while trunk lesions were found in 50.6%, mainly in the abdomen (27.0%). Upper limb lesions were present in 22.5%. A median of 5 lesions (IQR 3–6) per patient were found and were mostly ulcerative (95.5%).

### CUA diagnosis

The median time between onset of skin lesions and diagnosis was 46 days (IQR 24–88). When standard X-rays were performed (57.3%), calcifications were identified in arteries (29.4%), arterioles (15.7%) or both (31.4%), or vessels with extravascular calcifications (17.6%). In 24 patients (27.0%) examined by CT-scan, calcifications were identified in 75% of them. 12 out of 18 patients (66.7%) had a pathological nuclear bone scan. Transcutaneous oxygen measurement was pathological in 9 out of 11 evaluated patients. Doppler ultrasound (53 patients, 59.6%) revealed mostly medial calcification sclerosis associated with non-significant stenosis.

A skin biopsy was performed in 60 patients (67.4%), more frequently among non-dialyzed cases (79.0% vs 64.3%), with an average number of 1.6 (±0.8) biopsies per patient, and confirmed the diagnosis in 65% of cases. A specialized calcium staining was performed for 33.3% of cases, revealing calcification of arterioles (53.3%), tissues (8.3%), or both (10%). The other findings were thrombosis (50%), fibro-intimal hyperplasia (20%) and panniculitis (72%).

### CUA treatments

Treatments used for CUA were wound care, intravenous Sodium Thiosulfate (STS), renal replacement therapy modification and nutritional support therapy (Table 4). Other treatments were discontinued, notably VKA, calcium supply and vitamin D. Median dosing of intravenous STS was 75 g per week, and the cumulative dose was higher for dialysis patients. Some treatments were scarcely used, and included, intra-lesional STS (1.1%), hyperbaric oxygen (2.3%), bisphosphonate (2.3%) and vitamin K supplementation (1.1%).

**Table 4:** Multimodal treatment strategy of CUA: initiation and modifications of treatments after CUA diagnosis

Treatment	N	Total CUA cases	N	Dialysis cases	N	Non dialysis cases
Wound care	89	88 (98.9%)	70	70 (100.0%)	19	18 (94.7%)
Antibiotherapy	89	69 (77.5%)	70	53 (75.7%)	19	16 (84.2%)
Discontinuation of VKA	65	46 (70.8%)	54	38 (70.4%)	11	8 (72.7%)
Discontinuation of active vitamin D	20	14 (70.0%)	17	11 (64.7%)	3	3 (100.0%)
Intravenous STS	89	58 (65.2%)	70	45 (64.3%)	19	13 (68.4%)
STS cumulative dose (g)	56	488 (300–750)	43	525 (300–750)	13	375 (225–900)
STS duration (week)	58	6 (4–10)	45	6 (4–10)	13	5 (3–12)
Renal Replacement Therapy modification	89	57 (64.0%)				
Increase of dialysis duration and/or frequency			70	41 (58.6%)		
Start of dialysis					19	16 (84.2%)
Switch from HD to HDF			70	13 (18.6%)		
Switch from HDF to HD			70	4 (5.7%)		
Use of citrate dialysate	89	6 (6.7%)	70	6 (8.6%)	19	0 (0.0%)
Discontinuation or lowering of oral calcium supply	53	31 (58.5%)	46	26 (56.5%)	7	5 (71.43%)
Nutritional support therapy	89	47 (52.8%)	70	35 (50.0%)	19	12 (63.2%)
Sevelamer	89	42 (47.2%)	70	31 (44.3%)	19	11 (57.9%)
Initiation or dose increase of Sevelamer	89	24 (27.0%)	70	15 (21.4%)	19	9 (47.4%)
Initiation or dose increase of Cinacalcet	89	31 (34.8%)	70	27 (38.6%)	19	4 (21.1%)
Discontinuation of native vitamin D	52	18 (34.6%)	40	12 (30.0%)	12	6 (50.0%)
Surgical debridement	89	22 (24.7%)	70	17 (24.3%)	19	5 (26.3%)
≥ 2 surgical debridement	89	9 (10.1%)	70	8 (11.4%)	19	1 (5.26%)
Amputation	89	15 (16.9%)	70	13 (18.6%)	19	2 (10.5%)
Lanthanum carbonate	89	15 (16.9%)	70	15 (21.4%)	19	0 (0.0%)
Initiation or dose increase of Lanthanum carbonate	89	7 (7.9%)	70	7 (10.0%)	19	0 (0.0%)
Negative pressure wound therapy	89	12 (13.5%)	70	8 (11.4%)	19	4 (21.1%)
Discontinuation of iron therapy	62	8 (12.9%)	54	7 (13.0%)	8	1 (12.5%)
Standard oxygen therapy	89	11 (12.4%)	70	9 (12.9%)	19	2 (10.5%)
Initiation or dose increase of statin	89	10 (11.2%)	70	8 (11.4%)	19	2 (10.5%)
Skin transplantation	89	8 (9.0%)	70	7 (10.0%)	19	1 (5.3%)
Parathyroidectomy	89	5 (5.6%)	70	5 (7.1%)	19	0 (0.0%)
Steroids						
Discontinuation or dose decrease of steroids	12	7 (58.3%)	10	6 (60.0%)	2	1 (50.0%)
Initiation or dose increase of steroids	12	2 (16.7%)	10	2 (20.0%)	0	0 (0.0%)
Local steroids treatement	89	12 (13.5%)	70	9 (12.9%)	19	3 (15.8%)

Median (IQR) or N (%). *HD* hemodialysis, *HDF* hemodiafiltration, *STS* sodium thiosulfate, *VKA* vitamin K antagonist

### CUA risk factors among dialyzed patients

Univariate logistic regression analysis (Table S2) revealed dialyzed CUA patients had significantly more diabetes mellitus (Odds Ratio (OR) 2.7), diabetes and/or hypertension associated nephropathy (OR 3.0), symptomatic peripheral vascular disease (OR 2.0), history of cardiac failure (OR 4.6) or of pathologic fracture (OR 2.4). They had increased adjusted serum calcium (OR 9.2), serum phosphate (OR 4.6), calcium phosphate product (OR 2.0), normalized iPTH (OR 1.1) and CRP (OR 1.4) in the 6 months prior to identification of lesions (respectively OR 2.2; 5.4; 2.0; 1.1 and 1.1). Insulin (OR 2.1) and Erythropoiesis-Stimulating Agent (OR 2.6) also increased this risk. On the contrary, dialysis dose (eKt/V) (OR 0.2) and hemoglobin level at onset of lesions (OR 0.7) were associated with lower odds.

By multivariate analysis (Table 5), risk factors independently associated CUA in dialyzed patients were obesity, coronary artery disease, weight loss over the last 6 months, serum phosphate increase within 6 months before diagnosis and VKA therapy. As lower odds were associated with serum albumin increase within the 6 months before onset of lesions (OR 0.2), serum albumin decrease was also a risk factor of CUA among the dialysis cases.

**Table 5:** Multivariate logistic regression analysis of risk factors of CUA in dialysis cases compared to matched dialysis controls

Parameter	OR (95% CI)	*p*-value
Body Mass Index, per 5 kg/m^2^ increase	1.56 (1.08–2.27)	0.02
Loss of weight within 6 months before diagnosis, per 1 kg increment	1.66 (1.22–2.26)	0.001
Coronary artery disease	5.52 (1.07–28.65)	0.04
Albumin variation between diagnosis and 6 months before, per 5 g/L increment	0.19 (0.05–0.70)	0.01
Serum phosphate (worst value within 6 months before onset of CUA), per 1 mmol/L increment	9.27 (1.70–50.68)	0.01
Vitamin K Antagonist	5.11 (1.29–20.29)	0.02

### CUA outcome

**Fig. 2:**
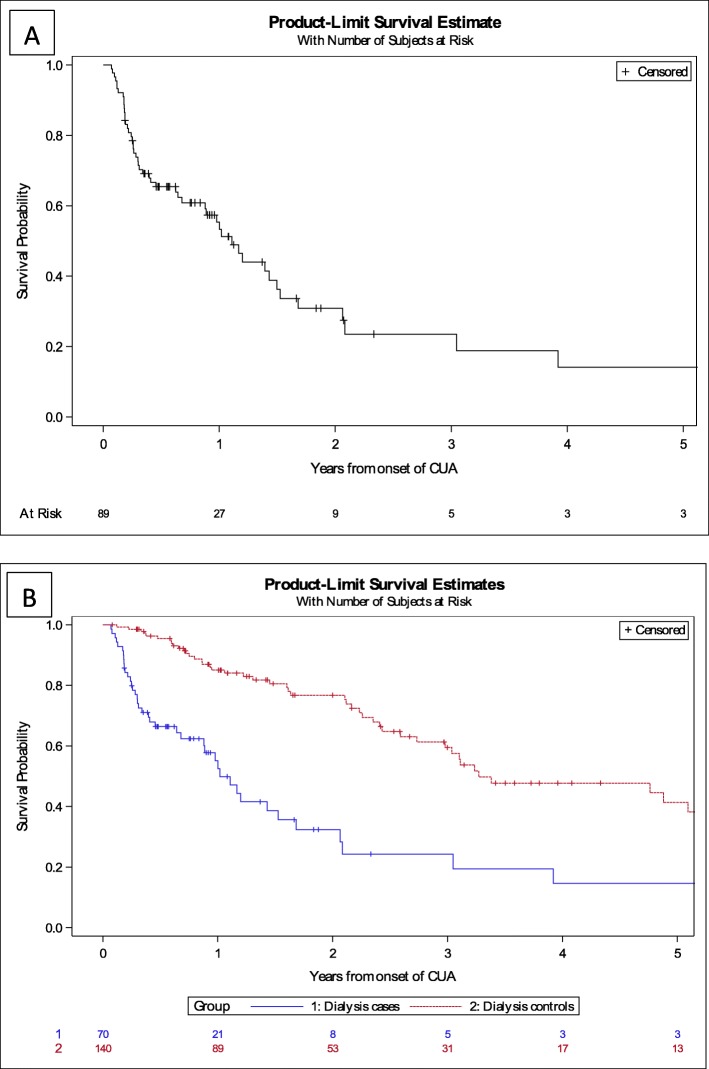
Survival among Calcific Uremic Arteriolopathy cases and the hemodialysis controls

40.4% of deaths due to calciphylaxis occurred during the first year after diagnosis and 56.2% after 5 years (Table 6). Mortality of dialysis cases was significantly higher than paired hemodialysis controls (Hazard Ratio (HR) 3.4; 95% Confidence Interval (CI) 2.2–5.2; *p* < 0.001) (Fig. 2). The median delay between the onset of lesions and death was 4.1 months (IQR 2.2–14.2). The main circumstance of death was in palliative care for CUA patients (49.1%).

**Table 6:** Evolution and outcome of CUA patients, compared to dialysis controls

	Total CUA cases (*N* = 89)	Non dialysis cases (*N* = 19)	Dialysis cases (*N* = 70)	Dialysis controls (*N* = 140)
Local evolution of CUA lesions				
Deterioration	37 (41.6%)	7 (36.4%)	30 (42.9%)	
Any improvement	2 (2.3%)	0 (0.0%)	2 (2.9%)	
Partial improvement	17 (19.9%)	5 (26.3%)	12 (17.1%)	
Complete healing	33 (37.1%)	7 (36.8%)	26 (37.1%)	
Reccurence of CUA	16 (31.4%)	2 (15.4%)	14 (36.8%)	
Crude mortality rate (uncensored)				
At 1 year after onset of lesions	36 (40.4%)	8 (42.1%)	28 (40.0%)	18 (12.9%)
At 2 years after onset of lesions	46 (51.7%)	10 (52.6%)	36 (51.4%)	25 (18.9%)
At 5 years after onset of lesions	50 (56.2%)	10 (52.6%)	40 (57.1%)	44 (31.4%)
Cause of death				
Cardiac arrest	9 (17.0%)	1 (10.0%)	8 (18.6%)	31 (49.2%)
Sepsis	9 (17.0%)	1 (10.0%)	8 (18.6%)	2 (3.2%)
Palliative care	26 (49.1%)	7 (70.0%)	19 (44.2%)	11 (17.5%)
Stroke	0 (0.0%)	0 (0.0%)	0 (0.0%)	3 (4.8%)
Cardiac failure	6 (11.3%)	0 (0.0%)	6 (11.3%)	0 (0.0%)
Death secondary to CUA	38 (71.7%)	7 (70.0%)	31 (72.1%)	0 (0.0%)

Complete healing of CUA lesions occurred in 37.1% (Table 6). The median delay between healing and diagnosis was 6.4 months (IQR 3.7–8.9). The median follow-up was respectively 6.1 (IQR 3.0–11.4) and 16.1 (IQR 8.7–36.1) months in dialysis cases and controls.

### Factors predictive of survival

Higher BMI (HR 0.79; *p* = 0.004), higher serum albumin at onset of lesions (HR 0.70; *p* < 0.001) and VKA discontinuation (HR 0.41; *p* = 0.01) were associated with better survival in univariate analysis only (Table S3). After removal of patients treated less than 2 weeks or with a cumulative dose below 150 g of STS, a trend to better survival was associated with STS cumulated dose and duration (HR 0.87; CI 0.77–0.97; *p* = 0.02).

By multivariate analysis, items with *p* < 0.2 in the univariate analysis were included, except sevelamer and lanthanum carbonate treatment at diagnosis, normalized PTH, eKt/V, number of skin biopsies, STS duration, VKA discontinuation and CUA recurrence, because of missing data. Adjusted serum calcium and calcium phosphate product were removed because of linkage to serum calcium and phosphate. Thus 192 patients were included in the multivariate analysis. Finally, factors independently associated with survival were surgical debridement (HR 0.11) and antibiotherapy (HR 0.25) (Table 7), whereas parathyroidectomy increased the risk of death (HR 29.5).

**Table 7:** Multivariate conditional logistic regression analysis of survival predictors among the 89 Calcific Uremic Arteriolopathy cases

Parameter	HR (95% CI)	*p*-value
Hemoglobin at diagnosis, per 1 g/dL increment	0.42 (0.30–0.60)	< 0.001
Insulin therapy	0.28 (0.12–0.65)	0.003
Lower limbs localization		< 0.001
Below knee VS none	0.13 (0.01–1.23)	0.20
Above knee VS none	0.17 (0.01–2.49)	0.72
Any localization VS none	107.04 (16.1–713)	< 0.001
Upper limbs localization		< 0.001
Below elbow VS none	10.79 (2.34–49.7)	0.002
Any localization VS none	267.48 (23.3–3069)	< 0.001
Type of CUA		< 0.001
Distal-type VS proximal-type	0.04 (0.00–0.44)	0.008
Proximal and distal type VS proximal type	0.01 (0.00–0.13)	< 0.001
Parathyroidectomy	29.53 (3.87–226)	0.001
Sevelamer	0.26 (0.10–0.66)	0.005
Lanthanum carbonate	0.04 (0.01–0.21)	< 0.001
Surgical debridement	0.11 (0.04–0.28)	< 0.001
Antibiotherapy	0.25 (0.08–0.73)	0.01
Local evolution of skin lesions		< 0.001
Partial improvement VS complete healing	1.14 (0.22–5.77)	0.88
Deterioration VS complete healing	497.78 (79.5–3118)	< 0.001
No improvement VS complete healing	112.48 (12.4–1023)	< 0.001

Hazard ratio (HR) with 95% confidence interval

## Discussion

The 89 CUA patients of our study were typically 70-year old overweight diabetic females under VKA therapy (72%). In these cases, bone mineral disease parameters were out of the recommended ranges and were associated with inflammation and malnutrition.

These patients were identified from all over western France and the informations provided by our study are the results of average clinical management of these patients and not the experience of one expert care center. By following the Hayashi criteria [8], a skin biopsy was not a prerequisite in our study and confirmed diagnosis only in required clinical situations, in particular, to rule out a differential diagnosis. Because this can worsen lesions, skin biopsies are frequently avoided. Specificity is also questioned because of the frequence of extravascular calcifications in ESRD. Skin biopsies can confirm diagnosis by showing the combination of arteriolar media calcification and thrombosis that is associated with CUA [9]. We think that the identified CUA cases are the reflect of complexity of CUA diagnosis in clinical practice.

The demographic data and CUA predisposing factors identified in our study were consistent with other case-control studies [4, 10]. Inflammation and bone mineral disease abnormalities, especially hyperphosphatemia and hyperparathyroidism, and malnutrition preceded CUA onset by months [2, 10]. For the first time, weight loss within the 6 months before CUA onset was identified by our study as a risk factor in dialysis patients.

The association of CUA and VKA therapy has already been reported [4, 8, 11]. By decreasing carboxylated matrix Gla Protein (cMGP), VKA are suspected to contribute to vascular calcification and therefore promote calciphylaxis. A low level of cMGP have also been highlighted in CUA cases associated with Vitamin K deficiency [12]. Besides, Warfarin could paradoxically favor thrombosis locally, by blocking protein S endothelial secretion in response to stress [13]. Thrombophilia is also a known risk factor of calciphylaxis [14, 15] and we assume that the low prevalence of thrombophilia in our study might be due to lack of systematic screening. As Direct oral anticoagulants have no pro-thrombotic effect and given that vessel thrombosis may play a key role in calciphylaxis, they have been used to replace VKA once diagnosis of CUA is confirmed [16, 17]. Two retrospective studies [16, 17] have assessed the safety of Apixaban in CUA patients: 4 bleeding events in 20 dialysis patients were found and a lower mortality rate was demonstrated. Additional comparative studies are of course necessary.

We were surprised to identify so many non-dialyzed patients with calciphylaxis. The frontier between uremic calciphylaxis and non-uremic calciphylaxis is difficult to define. Studies on non-uremic calciphylaxis are mainly case reports and case series. Interestingly, a review on Non Nephrogenic Calciphylaxis (NNC), defined as calciphylaxis occurring in patients without impaired renal function (eGFR > 60 mL/min/1.73m^2^), showed that VKA and obesity were the two main conditions associated with NNC [18]. Mean blood mineral parameters were normal. Calciphylaxis seems to be the conjunction of multiple conditions (obesity, VKA, bone mineral disease abnormalities, uremia, inflammation) with a broad spectrum of variations. In our study, non-dialyzed CUA patients were more obese and inflammation prior CUA onset was more severe than in dialyzed CUA patients. A link between arteriolar calcification and adipocytes could explain the preferential localization of calciphylaxis in adipose tissue areas and the increased risk of calciphylaxis associated with obesity [4, 19]. Childhood obesity is already known to increase coronary artery calcification by middle age [20]. An increase in NNC reported cases [18, 19] might not only be secondary to increased medical awareness of this disease, but also might be linked to the epidemic of obesity. In our study, non-dialyzed cases had the same survival as dialysis cases, whereas some studies have reported a better prognosis of NNC [21].

The mortality rate was particularly high in our study. Ulcerative lesions and proximal-type CUA, known as poor prognosis factors [2, 3], were predominant. The prolonged diagnosis delay (46 vs 28 days in the German registry [22]) could be explained by the use of ulcerative skin lesions as inclusion criteria, because non-ulcerative lesions (plaques, nodules) precede the ulcerative lesions by several days [3]. This diagnosis delay could also be linked to a lack of acknowledgment of early CUA lesions, as seen in the Japanese case control study [10]. The dialysis vintage, shorter among the dialyzed cases than the matched controls (1.4 vs 2.2 years) could not explain the excess of mortality in dialyzed cases. So calciphylaxis does not necessarily occur after a long dialysis vintage. This is consistent with the important number of CUA cases identified among patients with CKD stage 4–5.

The main circumstance of death in our study was palliative care (49%), in the absence of efficient therapy to treat CUA. Due to its promising treatment properties [23, 24], STS was frequently administered, but was only associated with a trend toward better prognosis after exclusion of patients treated less than 2 weeks. Trials are in progress to assess the real benefit of STS in CUA. As demonstrated by other studies, surgical debridement had a net impact on survival [25–27] and should be proposed more widely. Contrary to other studies [26], parathyroidectomy was associated with a poorer prognosis, but given that only 5 parathyroidectomies were performed the conclusions are limited.

## Conclusions

Our study confirms the data reported by others on CUA but showed for the first time the contribution of significant unintentional weight loss. Few therapeutic measures seem efficient. Among them, STS is commonly used, but its benefit has still to be proved. The ongoing trials are of major interest.

## Supplementary information

**Additional file 1: Table S1.** Differential diagnosis idendified among eligible patients. **Table S2.** Univariate logistic regression analysis of risk factors of CUA in dialysis cases compared to matched dialysis controls. **Table S3.** Univariate conditional logistic regression analysis of survival predictors among Calcific Uremic Arteriolopathy cases.

## Data Availability

All data generated or analysed during this study are included in this published article and its supplementary information files.

## Abbreviations

BMI: Body Mass Index
CKD: Chronic Kidney Disease
cMGP: carboxylated Matrix Gla Protein
CRP: C-Reactive Protein
CUA: Calcific Uremic Arteriolopathy
eGFR: estimated Glomerular Filtration Rate
ESRD: End Stage Renal Disease
HR: Hazard Ratio
iPTH: intact Parathyroid Hormone
IQR: Interquartile Range
NNC: Non Nephrogenic Calciphylaxis
OR: Odds Ratio
STS: Sodium Thiosulfate
VKA: Vitamin K antagonist
