# Supplementary material for: Calciphylaxis epidemiology, risk factors, treatment and survival among French chronic kidney disease patients: a case-control study
Source: BMC Nephrol. 2020 Feb 26;21:63. doi: 10.1186/s12882-020-01722-y (PMC7045437; doi:10.1186/s12882-020-01722-y)
Supplement: Supplementary file 1 — Additional file 1: Table S1. Differential diagnosis idendified among eligible patients. Table S2. Univariate logistic regression analysis of risk factors of CUA in dialysis cases compared to matched dialysis controls. Table S3. Univariate conditional logistic regression analysis of survival predictors among Calcific Uremic Arteriolopathy cases. [file 12882_2020_1722_MOESM1_ESM.pdf]

Table S1. Differential diagnosis idendified among eligible patients.

	n
Atherosclerotic vascular disease	6
Calcinosis cutis	3
Non uremic calciphylaxis	2
Pressure sore	2
Pyoderma gangrenosum	2
Necrotizing vasculitis	1
Cholesterol embolization	1
Warfarin-induced skin necrosis	1
Cryoglobulinemia skin vasculitis	1
Ischemic steal syndrome related to arteriovenous fistula	1
Acute febrile neutrophilic dermatosis (Sweet syndrome)	1
Perforating ulcers	1
Total	22

Table S2. Univariate logistic regression analysis of risk factors of CUA in dialysis cases compared to matched dialysis controls.

Parameter	N	OR (95% CI)	p-value
Age	210	1.25 (1.04-1.51)	0.02
Gender	210	1.00 (0.05-18.91)	1.00
BMI (per 5 kg/m^2 increase)	207	1.65 (1.30-2.09)	<0.001
BMI clusters (kg/m^2)	207		<0.001
Underweight vs Normal (18.5-25)		0.39 (0.08-2.01)	0.35
Overweight (25-30) vs Normal (18.5-25)		1.03 (0.43-2.47)	0.87
Obesity (30-40) vs Normal (18.5-25)		2.93 (1.23-6.96)	0.01
Severe obesity (> 40) vs Normal (18.5-25)		12.78 (3.29-49.65)	<0.001
Loss of weight within 6 months before diagnosis	200	1.45 (1.24-1.69)	<0.001
Diabetic and/or hypertension related nephropathy	210	3.00 (1.64-5.47)	<0.001
Nephropathy	210		0.054
Diabetes-associated nephropathy		6.90 (1.61-29.64)	0.01
Hypertension-associated nephropathy		4.89 (1.18-20.27)	0.03
Hypertension and diabetes-associated nephropathy		3.25 (0.76-13.85)	0.11
Glomerular nephropathy (diabetic excluded)		1.54 (0.35-6.70)	0.56
ADPKD		0.79 (0.11-5.48)	0.81
Tubulo-interstitial nephropathy		1.75 (0.38-8.08)	0.47
Other nephropathy		1.16 (0.10-13.82)	0.91
Multiple causes		3.28 (0.65-16.64)	0.15
History of PD	210	1.78 (0.63-5.08)	0.28
In-center dialysis	207	2.12 (0.74-6.06)	0.16
History of kidney graft	210	0.54 (0.21-1.39)	0.20
CAD	210	1.94 (1.04-3.62)	0.04
Heart failure	210	4.63 (2.32-9.26)	<0.001
Stroke	210	0.86 (0.39-1.86)	0.70
PAD with symptoms	210	1.95 (1.07-3.53)	0.03
Diabetes mellitus	210	2.71 (1.40-5.27)	0.003
Arterial Hypertension	210	3.47 (0.99-12.14)	0.05
Hypercholesterolemia	210	0.87 (0.49-1.54)	0.62
Parathyroidectomy	210	1.00 (0.30-3.32)	1.00
History of pathologic fracture	210	2.41 (1.11-5.27)	0.03
History of smoking	207	1.23 (0.57-2.62)	0.60
Progressive cancer	210	1.00 (0.38-2.66)	1.00
Hepatobiliary disease	210	1.78 (0.79-3.98)	0.16
Chronic alcoholism	210	1.77 (0.50-6.24)	0.38
Auto-immune disorder (connective tissue disease)	210	2.00 (0.58-6.91)	0.27
Thrombophilia	210	1.25 (0.41-3.82)	0.70
**At onset of lesions**			
Total serum calcium, per 0.5 mmol/L increment	208	2.63 (1.12-6.15)	0.03
Adjusted serum calcium, per 0.5 mmol/L increment	206	9.16 (3.77-22.25)	<0.001
Serum phosphate, per 1 mmol/L increment	208	4.57(2.36-8.85)	<0.001
Calcium phosphate product, per 1 mmol2/L2 increment	210	2.03 (1.52-2.73)	<0.001
Serum Albumin, per 5 g/L increment	206	0.82 (0.76-0.89)	<0.001
Serum Albumin variation between diagnosis and 6 months before, per 5 g/L increment	202	0.40 (0.27-0.61)	<0.001

iPTH (pg/mL)	206	1.00 (1.00-1.00)	0.16
Normalized iPTH, per 1 N increment	206	1.07 (1.01-1.12)	0.02
iPTH outside of target range between 2 and 9 fold normal range	210	1.44 (0.79-2.62)	0.23
25-Hydroxyvitamin D (ng/mL)	156	1.00 (0.98-1.01)	0.63
CRP, per 10 mg/L increment	205	1.37 (1.17-1.59)	<0.001
Hemoglobin, per 1 g/dL increment	206	0.72 (0.58-0.90)	0.005
Hemoglobin A1c, per 1% increment	90	1.35 (0.82-2.22)	0.23
**Worst value within 6 months before onset of CUA**			
Total serum calcium, per 0.5 mmol/L increment	206	164.17 (0.07-383010.8)	0.20
Adjusted serum calcium, per 0.5 mmol/L increment	203	2.15 (1.09-4.27)	0.03
Serum phosphorate, per 1 mmol/L increment	206	5.43 (2.58-11.41)	<0.001
Calcium phosphate product, per 1 mmol2/L2 increment	206	2.00 (1.48-2.70)	<0.001
Serum Albumin, per 5 g/L increment	203	0.94 (0.89-1.00)	0.05
iPTH (pg/mL)	193	1.00 (1.00-1.00)	0.08
Normalized iPTH, per 1 N increment	193	1.06 (1.01-1.11)	0.01
iPTH outside of target range between 2 and 9 fold normal range	210	2.02 (1.10-3.71)	0.02
25-Hydroxyvitamin D (ng/mL)	151	0.99 (0.97-1.01)	0.26
CRP, per 10 mg/L increment	200	1.10 (1.03-1.16)	0.002
**Medication**			
25-hydroxyvitamin D	207	0.82 (0.44-1.51)	0.53
Active vitamin D	207	0.98 (0.48-2.00)	0.95
Calcium-based phosphate binders	207	1.69 (0.97-2.93)	0.06
Non calcium-based phosphate binders	207	1.36 (0.72-2.58)	0.34
Sevelamer	210	1.29 (0.70-2.36)	0.41
Lanthanum carbonate	210	1.18 (0.54-2.58)	0.68
Cinacalcet	206	1.23 (0.62-2.46)	0.55
Betablocker	207	1.39 (0.78-2.50)	0.27
Insulin therapy	208	2.12 (1.08-4.14)	0.03
Vitamin K Antagonist	208	8.42 (3.93-18.02)	<0.001
Corticosteroids	208	1.00 (0.39-2.60)	1.00
Statin	208	1.08 (0.60-1.94)	0.80
ESA	207	2.56 (1.06-6.14)	0.04
Iron therapy	207	1.27 (0.62-2.61)	0.51
ACEi/ARB	208	1.14 (0.62-2.10)	0.67
Dialysis method: HD vs HDF	205	1.08 (0.55-2.12)	0.82
eKt/V	193	0.18 (0.07-0.46)	<0.001
Citrate buffer	210	1.00 (0.36-2.74)	1.00

ACEi/ARB, angiotensin converting enzyme inhibitor/angiotensin receptor blocker; ADPKD, autosomal dominant polycystic kidney disease; BMI, body mass index; CAD, coronary artery disease; CKD, chronic kidney disease; CRP, C-reactive protein; CUA, calcific uremic arteriolopathy; ESA, erythropoiesis-stimulating agent; iPTH, intact parathyroid hormon; PAD, peripheral artery disease; PD, peritoneal dialysis.

Table S3. Univariate conditional logistic regression analysis of survival predictors among Calcific Uremic Arteriolopathy cases.

Parameter	N	HR	p-value
Age	89	1.02 (0.99-1.05)	0.19
Gender	89	0.91 (0.52-1.59)	0.73
BMI (per 5 kg/m^2 increase)	89	0.79 (0.67-0.93)	0.004
BMI clusters (kg/m^2)	89		0.10
Underweight vs Normal (18.5-25)		0.78 (0.18-3.44)	
Overweight (25-30) vs Normal (18.5-25)		0.80 (0.37-1.75)	
Obesity (30-40) vs Normal (18.5-25)		0.59 (0.29-1.21)	
Severe obesity (> 40) vs Normal (18.5-25)		0.25 (0.09-0.71)	
Loss of weight within 6 months before diagnosis	82	1.03 (0.99-1.06)	0.11
Nephropathy	89		0.37
Diabetes-associated nephropathy vs Unknown nephropathy		2.47 (0.55-11.1)	0.24
Hypertension-associated nephropathy		1.91 (0.39-9.38)	0.43
Hypertension and diabetes-associated nephropathy		3.89 (0.83-18.2)	0.08
Glomerular nephropathy (diabetic excluded)		1.59 (0.29-8.55)	0.59
ADPKD		2.39 (0.32-17.6)	0.39
Tubulo-interstitial nephropathy		1.88 (0.33-10.7)	0.48
Other nephropathy		6.68 (0.58-77.4)	0.13
Multiple causes		5.25 (0.96-28.7)	0.06
History of PD	89	0.68 (0.29-1.61)	0.38
In-center dialysis	67	1.48 (0.45-4.88)	0.52
History of kidney graft	89	0.57 (0.20-1.62)	0.29
CAD	89	1.01 (0.59-1.75)	0.96
Heart failure	89	1.45(0.81-2.57)	0.21
Stroke	89	1.19 (0.59-2.39)	0.63
PAD with symptoms	89	1.15 (0.66-2.02)	0.63
Diabetes mellitus	89	1.24 (0.70-2.21)	0.47
Arterial Hypertension	89	0.93 (0.33-2.60)	0.88
Hypercholesterolemia	89	0.87 (0.49-1.55)	0.64
Parathyroidectomy	89	0.39 (0.05-2.87)	0.36
History of pathologic fracture	89	0.89 (0.45-1.74)	0.73
History of smoking	89	0.67 (0.33-1.36)	0.27
Progressive cancer	89	1.19 (0.50-2.82)	0.69
Hepatobiliary disease	89	1.02 (0.51-2.02)	0.96
Chronic alcoholism	89	0.55 (0.19-1.57)	0.26
Auto-immune disorder (connective tissue disease)	89	0.56 (0.14-2.30)	0.42
Thrombophilia	89	0.30 (0.04-2.21)	0.24
**At onset of lesions**			
Total serum calcium, per 0.5 mmol/L increment	89	0.74 (0.40-1.39)	0.35
Adjusted serum calcium, per 0.5 mmol/L increment	88	1.59 (0.85-2.97)	0.15
Serum phosphate, per 1 mmol/L increment	89	0.82 (0.55-1.23)	0.34
Calcium phosphate product, per 1 mmol2/L2 increment	89	0.89 (0.74-1.07)	0.22
Serum Albumin, per 5 g/L increment	88	0.70 (0.58-0.84)	<0.001
Serum Albumin variation between diagnosis and 6 months before, per 5 g/L increment	80	0.77 (0.62-0.96)	0.02
iPTH (pg/mL)	87	1.00 (1.00-1.00)	0.69

Normalized iPTH, per 1 N increment	87	0.99 (0.95-1.03)	0.71
iPTH outside of target range between 2 and 9 fold normal range	89	0.83 (0.48-1.44)	0.50
25-Hydroxyvitamin D (ng/mL)	68	0.99 (0.97-1.01)	0.33
CRP, per 10 mg/L increment	87	1.03 (0.99-1.07)	0.12
Hemoglobin, per 1 g/dL increment	89	0.85 (0.70-1.04)	0.11
Hemoglobin A1c, per 1% increment	54	1.30 (0.97-1.74)	0.08
**Worst value within 6 months before onset of CUA**			
Total serum calcium, per 0.5 mmol/L increment	86	18.13 (0.03-9669)	0.37
Adjusted serum calcium, per 0.5 mmol/L increment	82	1.68 (0.96-2.94)	0.07
Serum phosphate, per 1 mmol/L increment	86	0.82 (0.56-1.19)	0.30
Calcium phosphate product, per 1 mmol2/L2 increment	86	0.93 (0.80-1.07)	0.30
Serum Albumin, per 5 g/L increment	81	0.86 (0.68-1.10)	0.22
iPTH (pg/mL)	75	1.00 (1.00-1.00)	0.22
Normalized iPTH, per 1 N increment	75	0.97 (0.94-1.01)	0.17
iPTH outside of target range between 2 and 9 fold normal range	89	0.82 (0.47-1.44)	0.49
25-Hydroxyvitamin D (ng/mL)	59	1.00 (0.98-1.01)	0.72
CRP, per 10 mg/L increment	80	1.02 (0.98-1.06)	0.30
**Medication**			
25-hydroxyvitamin D	89	0.98 (0.55-1.72)	0.94
Active vitamin D	89	0.72 (0.37-1.40)	0.33
Calcium-based phosphate binders	89	0.67 (0.38-1.17)	0.16
Non calcium-based phosphate binders	89	0.36 (0.20-0.64)	<0.001
Sevelamer	89	0.61 (0.34-1.10)	0.10
Lanthanum carbonate	89	0.25 (0.08-0.79)	0.02
Cinacalcet	89	1.43 (0.72-2.81)	0.31
Betablocker	89	0.76 (0.44-1.32)	0.33
Insulin therapy	89	1.45 (0.82-2.56)	0.20
Vitamin K Antagonist	89	0.90 (0.49-1.65)	0.73
Corticosteroids	89	1.62 (0.72-3.64)	0.24
Statin	89	1.05(0.60-1.84)	0.87
ESA	89	1.19 (0.58-2.45)	0.64
Iron therapy	89	1.09(0.59-2.03)	0.79
ACEi/ARB	89	0.70 (0.39-1.27)	0.24
Dialysis method: HD vs HDF	67	1.16 (0.58-2.31)	0.68
eKt/V	62	1.00 (0.38-2.66)	0.99
Citrate buffer	89	1.85 (0.77-4.42)	0.17
**Triggering event within the three months before onset**			
Local triggering event	28		0.49
Subcutaneous injection of heparin VS local trauma		1.37 (0.37-5.05)	0.64
Insulin injection VS local trauma		0.69 (0.20-2.40)	0.56
Subcutaneous injection of heparin and insulin injection VS local trauma		2.02(0.35-11.7)	0.43
Hypovolemia	89	1.36 (0.78-2.38)	0.28
Time between beginning of dialysis and onset of CUA	70	1.00 (1.00-1.00)	0.73
Diagnosis delay, per 1 week increment	88	0.97 (0.95-1.00)	0.054
**Localization**			
Lower limbs localization	89		0.08
Below knee VS none		1.48 (0.50-4.40)	0.48
Above knee VS none		2.73 (0.92-8.16)	0.07

Any localization VS none		2.93 (0.93-9.27)	0.07
Trunk localization	89		0.54
Gentiale localization VS none		1.71 (0.51-5.71)	0.39
Hip and buttock VS none		1.54 (0.46-5.16)	0.49
Multiple trunck localization VS none		1.31 (0.58-2.95)	0.51
Abdomen VS none		1.76 (0.89-3.45)	0.10
Upper limbs localization	89		0.12
Below elbow VS none		0.46 (0.14-1.52)	0.46
Any localization VS none		1.60 (0.63-4.11)	0.32
Type of CUA	89		0.049
Distal-type VS proximal-type		0.57 (0.29-1.15)	0.12
Proximal and distal type VS proximal type		1.42 (0.74-2.73)	0.29
Number of skin lesions	89	1.03 (0.94-1.14)	0.54
**Description of lesion**			
Reticulate purpura or livedo reticularis	89	0.45 (0.24-0.86)	0.02
Eschar	89	1.26 (0.50-3.21)	0.63
Violaceous plaque	89	1.60 (0.89-2.88)	0.11
Ulceration	89	1.12 (0.27-4.62)	0.88
**Skin biopsy**			
Skin biopsy performed	89	0.67 (0.37-1.22)	0.19
Number of skin biopsy performed	60	0.53 (0.29-0.95)	0.03
Skin biopsy performed by a surgeon	60	1.58 (0.66-3.74)	0.30
**Histopathological findings**			
Calcification in histopathological examination	60		0.05
Arteriole VS none		1.30 (0.57-2.95)	0.54
Arteriole and extravascular VS none		3.50 (1.11-11.0)	0.03
Extravascular VS none		0.22 (0.03-1.78)	0.16
Thrombosis	60	1.51 (0.76-3.01)	0.24
Intimal fibrosis	60	0.72 (0.31-1.64)	0.43
Panniculitis	60	0.69 (0.34-1.42)	0.32
**Treatments**			
Intravenous STS	89	0.69 (0.40-1.19)	0.18
STS cumulative dose (g)	56	1.00 (0.98-1.02)	0.78
STS duration (week)	58	0.88 (0.80-0.96)	0.005
Effective Intravenous STS *			
STS cumulative dose (g), per 100g increment	51	0.87 (0.77-0.97)	0.02
STS duration (week)	51	0.87 (0.77-0.97)	0.02
Cinacalcet	89		0.27
Initiation or dose increase VS none		0.66 (0.36-1.21)	0.18
Continuation VS none		1.33 (0.51-3.46)	0.55
Parathyroidectomy	89	0.38 (0.11-1.25)	0.11
Sevelamer	89		0.03
Initiation or dose increase VS none		1.49 (0.79-2.82)	0.22
Continuation VS none		0.38 (0.15-0.94)	0.04
Lanthanum carbonate	89		0.14
Initiation or dose increase VS none		0.64 (0.23-1.81)	0.40
Continuation VS none		0.26 (0.06-1.10)	0.07
Statin	89		0.40

Initiation or dose increase VS none		0.45 (0.13-1.50)	
Continuation VS none		1.01 (0.56-1.80)	
Oxygen therapy : Standard oxygen therapy VS none	89	0.83 (0.37-1.86)	0.90
Surgical debridement	89	0.66 (0.35-1.24)	0.20
Amputation	89	0.99 (0.50-1.97)	0.98
Skin transplantation	89	0.49 (0.17-1.41)	0.19
Negative pressure wound therapy	89	0.91 (0.41-2.02)	0.81
**Renal Replacement Therapy modification**			
Increase of dialysis duration and/or frequency	89	1.02 (0.58-1.81)	0.94
Hemodialysis method	86		0.56
Switch from HD to HDF VS no modification		1.12 (0.43-2.90)	0.81
Switch from HDF to HD VS non modification		0.71 (0.36-1.40)	0.32
Use of citrate dialysate	89	1.72 (0.61-4.80)	0.30
Nutritional support therapy	89	1.46 (0.83-2.57)	0.19
Antibiotherapy	89	0.60 (0.32-1.13)	0.11
Oral calcium supply	53		0.23
Increase VS discontinuation		1.23 (0.27-5.72)	0.79
Lowering VS discontinuation		0.71 (0.19-2.58)	0.60
No modification VS discontinuation		1.95 (0.90-4.23)	0.09
Discontinuation of VKA	65	0.41 (0.21-0.82)	0.01
Discontinuation of iron therapy	62	1.66 (0.68-4.03)	0.26
Steroids	12		0.99
Discontinuation VS no modification		0.87 (0.13-5.79)	0.88
Dose decrease VS no modification		1.11 (0.14-8.69)	0.92
Initiation or dose increase VS no modification		1.15 (0.16-8.38)	0.89
Local steroids treatement	89	0.95(0.44-2.03)	0.89
Discontinuation of native vitamin D	52	1.24 (0.57-2.71)	0.59
Discontinuation of active vitamin D	20	2.14 (0.46-9.92)	0.33
Local evolution of skin lesions	89		<0.001
Partial improvement VS complete healing		1.32(0.46-3.84)	0.61
Deterioration VS complete healing		11.45 (4.84-27.1)	<0.001
No improvement VS complete healing		35.04 (6.51-189)	<0.001

Hazard ratio (HR) with 95% confidence interval. ACEi/ARB, angiotensin converting enzyme inhibitor/angiotensin receptor blocker; ADPKD, autosomal dominant polycystic kidney disease; BMI, body mass index; CAD, coronary artery disease; CKD, chronic kidney disease; CRP, C-reactive protein; CUA, calcific uremic arteriolopathy; ESA, erythropoiesis-stimulating agent; HD, hemodialysis; HDF: hemodiafiltration; iPTH, intact parathyroid hormon; PAD, peripheral artery disease; PD, peritoneal dialysis; STS, sodium thiosulfate; VKA, vitamin K antagonist. *After removal of patients treated with intravenous STS for less than 2 weeks or with a cumulative dose of less than 150 g.
